# FALLING FOR FADS? Diagnostic and therapeutic fads in Psychiatry

**DOI:** 10.1192/j.eurpsy.2022.2290

**Published:** 2022-09-01

**Authors:** S. Jesus, A. Costa, G. Simões, J. Alcafache, P. Garrido

**Affiliations:** Baixo Vouga Hospital Centre - EPE, Psychiatry And Mental Health Department, Aveiro, Portugal

**Keywords:** trends, diagnostics and therapeutics, classification

## Abstract

**Introduction:**

Psychiatry is not immune to the effects of trends and fads, which are ideas that elicit short-lived enthusiasm, are quickly adopted, and abandoned when they fail to live up to expectations. Trends meet a deeply felt need to explain, or at least name, what would alternatively be unexplainable human suffering.

**Objectives:**

The authors aim to explore the trends and fads that have occurred in psychiatric diagnostic and treatment throughout history and assess if any modern trends can be identified as well as assessing the effects or consequences of these.

**Methods:**

The authors conducted a non-systematized literature review with focus on those articles most pertinent to the topic in question.

**Results:**

The literature demonstrates that fads and trends not only plague fashion and diet, but also psychiatry. Trends in psychotherapeutic options can be observed by the swing from psychoanalysis to psychopharmacological focus. Overdiagnosis is one of the consequences of these trends, and can be seen from hysteria, schizophrenia, multiple personality disorder, attention-deficit and hyperactivity disorder to gender identity disorder. These trends impact the way diagnosis are made and the treatments implemented.

**Conclusions:**

Fads in psychiatry have occurred not only on the edge, but in the very mainstream of theory and practice. A balance is called for, with caution needed in order not to fall into the temptation of the fad, however, an open mind should also be maintained when cutting-edge treatments and theories emerge. The sensible antidote to falling for fads and trends in psychiatry is commitment to evidence-based medicine.

**Disclosure:**

No significant relationships.

